# SGLT2 Inhibitors as Potential Anticancer Agents

**DOI:** 10.3390/biomedicines11071867

**Published:** 2023-06-30

**Authors:** Debasish Basak, David Gamez, Subrata Deb

**Affiliations:** Department of Pharmaceutical Sciences, College of Pharmacy, Larkin University, Miami, FL 33169, USA

**Keywords:** sodium-glucose cotransporter 2, anticancer, in vitro, in vivo, clinical

## Abstract

Sodium-glucose cotransporter 2 (SGLT2) serves as a critical glucose transporter that has been reported to be overexpressed in cancer models, followed by increased glucose uptake in both mice and humans. Inhibition of its expression can robustly thwart tumor development in vitro and in vivo. SGLT2 inhibitors are a comparatively new class of antidiabetic drugs that have demonstrated anticancer effects in several malignancies, including breast, liver, pancreatic, thyroid, prostate, and lung cancers. This review aims to assess the extent of SGLT involvement in different cancer cell lines and discuss the pharmacology, mechanisms of action, and potential applications of SGLT2 inhibitors to reduce tumorigenesis and its progression. Although these agents display a common mechanism of action, they exhibit distinct affinity towards the SGLT type 2 transporter compared to the SGLT type 1 transporter and varying extents of bioavailability and half-lives. While suppression of glucose uptake has been attributed to their primary mode of antidiabetic action, SGLT2 inhibitors have demonstrated several mechanistic ways to combat cancer, including mitochondrial membrane instability, suppression of β-catenin, and PI3K-Akt pathways, increase in cell cycle arrest and apoptosis, and downregulation of oxidative phosphorylation. Growing evidence and ongoing clinical trials suggest a potential benefit of combination therapy using an SGLT2 inhibitor with the standard chemotherapeutic regimen. Nevertheless, further experimental and clinical evidence is required to characterize the expression and role of SGLTs in different cancer types, the activity of different SGLT subtypes, and their role in tumor development and progression.

## 1. Introduction 

Cancer is a major public health concern, with the incidence and mortality from this lethal disease rapidly growing throughout the world. In 2020, about 19.3 million cancer cases were diagnosed, and 10 million deaths from cancer were reported worldwide [1]. Cancer also imposes a significant financial burden on families, and hence, it’s critical to develop novel agents that can effectively eradicate tumors. About a century ago, the “Warburg effect” postulated that malignant cells consume more glucose than normal cells, a theory that later became the basis that cancer is a metabolic disease [2]. Glucose serves as a significant energy source for cancer cells to meet their biosynthetic needs and to adapt to various microenvironments. Reprogramming of glucose metabolism results in several hallmark features of cancer, such as accelerated cell proliferation, angiogenesis, metastasis, and evasion of apoptosis [3]. An elevated level of glucose or hyperglycemia poses a risk factor for cancer and can result in increased tumor volume and growth in vivo [4]. Excess glucose is associated with increased sex steroid synthesis, vascular endothelial growth factor production, and oxidative stress, which are well-established risk factors for tumorigenesis [5,6]. Sugar promotes insulin resistance and upregulates the level of insulin-like growth factors, which in turn increases the risk of cancer by stimulating tumor cell growth and migration as well as upregulating angiogenesis [7]. In fact, epidemiological studies reported a correlation between diabetes mellitus and several cancers. Hyperglycemia, hyperinsulinemia, genetic factors, inflammation, and oxidative stress notably contribute to the crosstalk between diabetes mellitus and cancers [8]. It is evident that sugar serves as a stimulant for tumor cell growth. Sodium-glucose cotransporter 2 (SGLT2) inhibitors are relatively newer antidiabetic agents that have been reported to furnish anticancer effects in various in vitro and in vivo models. It is already well-established that certain types of tumor cells express SGLT2 transporters [9,10]. The rationale behind using these drugs in cancer models aligns well since cancer cells are heavily dependent on glucose utilization for their growth and proliferation. Apart from their usual mechanism of glucose uptake inhibition, these drugs also induced cell cycle arrest and apoptosis and destabilized mitochondrial membrane potential [11]. In fact, more mechanistic aspects of these agents are evolving with time.

Currently, available chemotherapeutics have expanded life expectancy, albeit with limited efficacy in different settings, and most importantly, they are not devoid of toxicities. Along with the induction of nausea, vomiting, and altering physical appearance, chronic effects of chemotherapy include drug resistance. Some likely causes include a change in membrane transport of the drug, mutated topoisomerase I, decreased drug activation, and augmented response against apoptosis due to mutated p53. Multidrug resistance is possible due to the efflux of drugs and genetic alterations leading to mutations and enhanced DNA repair capacity [12]. As a result, the efficacy of chemotherapy gets decreased resulting in a need for more focused cancer treatment. Given the study reports of SGLT2 expression in different cancers and its potential role in tumor growth, the antidiabetic drug class SGLT2 inhibitors have been explored in various in vitro and in vivo tumor models. These agents showed promising results in such models, and it is conceivable that limiting glucose supply to cancer cells could negatively impact tumor growth and proliferation. In this review, we aimed to delineate the function of SGLT2 in tumorigenesis and its progression, its mechanisms of action, and the potential applications of different SGLT2 inhibitors as anticancer agents.

## 2. Role of Glucose and Its Transporters in Cancer

**Figure 1:**
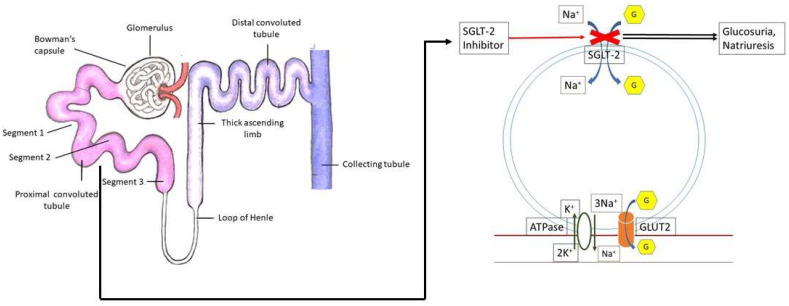
Mechanism of SGLT2 inhibitors in reducing glucose reabsorption.

SGLT2 is expressed in the proximal tubule of the kidneys, where this transporter is used to recover glucose from the filtrate. The retrieval of glucose from the glomerular filtrate is implemented by SGLT2 and is unrelated to insulin action. Approximately 90% of filtered glucose from the kidneys undergo SGLT2-mediated reabsorption in the first segment (S1) of the proximal convoluted tubule, while the remaining 10% gets reabsorbed by SGLT1 in the distal segment (S3). SGLT1 is mainly expressed in the small intestine and is also found in the liver, albeit at a lower level. SGLTs use the energy from a sodium/potassium ATPase pump to transport glucose against its concentration gradient (Figure 1). Glucose transporters (GLUTs) are other major glucose transporter proteins, which are encoded by solute carrier family 2 (SLC2A) genes, GLUTs function by passively allowing glucose to enter the cell membrane down its concentration gradient until both sides reach equilibrium. GLUT1 and GLUT2 play a role in reabsorbing glucose into the bloodstream in the proximal convoluted tubule [13]. GLUT overexpression in prostate cancer and other tumors is well studied, and an elevated expression of hypoxia-inducible factor 1 leads to the expression of GLUT1 and GLUT3 that upregulate glucose uptake and drives glucose to the glycolytic pathway [14,15]. Thus, the glucose metabolic reprogramming in tumor cells induces both the expression and translocation of GLUTs and other glycolytic enzymes to the plasma membrane [16].

Energy is indispensable for optimum growth and proliferation of cells. Usually, normal cells depend on the tricarboxylic acid cycle for energy; however, cancer cells heavily rely on glycolysis [17]. Thus, hyperglycemia provides cancer cells with a more robust environment to survive and proliferate. Concomitantly, a high level of blood glucose facilitates the synthesis of tumor protein and DNA that would otherwise stimulate tumor growth and metastasis [18]. Moreover, hyperglycemia is linked to the generation of reactive oxygen species (ROS) that may facilitate the production of advanced glycation end products (AGEs) [19]. ROS can lead to irreversible DNA damage, thereby inducing genetic mutation. This ultimately results in tumor metastasis through the activation of mitogen-activated protein (MAP) kinase and p21-activated kinase [20]. AGEs are linked to chronic inflammation that might be critical to induce genetic mutation and evolution and resulting in advanced stages of cancer [21]. Chronic hyperinsulinemia lowers the levels of sex hormone-binding globulin (SHBG). This results in the availability of plenty of sex hormones, such as estrogen and testosterone, which can increase the likelihood of hormonal-sensitive cancers like postmenopausal breast or prostate cancers [22]. Apart from its direct role in tumor cell proliferation and metastasis, elevated glucose can also induce an inflammatory state that has the potential to alter the tumor microenvironment producing an excess of cytokines such as interleukin 6 (IL-6), tumor necrosis factor alpha (TNF-α), and vascular endothelial growth factor (VEGF) [23]. This alteration in the cytokine milieu elicited by chronic inflammation may serve as the rudimentary stage for tumor development and progression. Hence, a heightened glucose level or diabetes is instrumental in the development of diabetes that would otherwise serve as a precursor to cancer.

## 3. Expression of SGLT2 in Cancerous Cells/Tissues

The expression of SGLT2 and its putative role in cancer was first reported by Ishikawa et al., who found that SGLT2 was significantly expressed in the liver and lymph nodes of metastatic lung cancer. These results led to the preliminary conclusion that SGLT2 played a critical role in glucose uptake in lung cancer metastasis [24]. Afterward, the landmark report by Scafoglio et al. that SGLT2 is functionally expressed in tumors paved the way for further research opportunities on SGLT2 inhibitors [10]. This research group was the first to demonstrate the expression of SGLT2 in pancreatic and prostate adenocarcinomas. The study showed the uptake of glucose in tumors by using SGLT-specific radioactive glucose analog, α-methyl4-deoxy-4-[18F]fluoro-D-glucopyranoside (Me4FDG). Most importantly, treatment with SGLT2 inhibitors (dapagliflozin and canagliflozin) decreased tumor growth and induced tumor necrosis [10]. In clear cell renal cell carcinoma, increased expression of SGLT2 was linked to a poor prognosis and a decrease in overall survival rates [9]. Another recent report demonstrated the presence of SGLT2 in cancerous tissues. In this study, human pancreatic and prostate adenocarcinomas revealed the presence of SGLT2, as evidenced by in vitro Me4FDG uptake assays and immunocytochemistry. The same transporter expression was found in mouse models, as shown by Me4FDG micro-PET (positron emission tomography), ex vivo autoradiography, and immunocytochemistry. The study also showed the uptake of Me4FDG in high-grade glioblastoma patients [25]. From these reports, it is conceivable that overexpression of SGLT2 transporter in tumors opens a potential avenue for targeted treatment with specific SGLT2 inhibitors.

Due to the exclusive specificity of 2-FDG for GLUT transporters [Km > 300 mM at 37 °C for SGLT2], a second tracer specific to SGLTs [Km < 6 mM] namely, α-methyl-4[^18^F]-4-deoxy-D-glucopyranose (Me4FDG) was developed. Me4FDG, unlike 2-FDG, did not cross the blood–brain barrier, accumulated intracellularly propelled by SGLT’s active transport mechanism, and is not excreted in the urine. Me4FDG PET imaging was employed on human tumor samples that were retrieved from operating rooms to reveal hot spots of Me4FDG uptake in pancreatic and prostate adenocarcinomas. The NSG xenograft mouse model demonstrated in vivo staining of mice pancreatic and prostate cell lines (ASPC-1/PC-3) through Me4FDG accumulation in vital regions of the tumors. Further, SGLT immunohistochemistry revealed robust SGLT2 staining in regions along the cell membranes and light staining of SGLT1; however, SGLT1 staining appeared exclusively on the cell nucleus [25].

## 4. Properties of SGLT2 Inhibitors

SGLT2 inhibitors share a common mechanism of action and pharmacodynamic profile but have varying pharmacokinetic properties, specifically in SGLT2/1 affinity, half-lives (t½), and bioavailability (F) (Table 1). The most recently approved agent, bexagliflozin, is over 90% plasma protein bound with a bioavailability of about 78% and T½ of 12 h. It is excreted mainly through glucuronidation by uridine 5′-diphospho-glucuronosyltransferase 1A9 (UGT1A9) and, to a lesser extent, by cytochrome P4503A (CYP3A) and the major metabolite is 3′-O-glucuronide [26]. Canagliflozin is also highly plasma protein bound (99%), with bioavailability and t½ of 65% and 12 h, respectively. It is metabolized by cytochrome P450 3A4 (CYP3A4). Dapagliflozin is 93% plasma protein bound, with a bioavailability of 78%. Its t½ is about 8–12 h and is excreted by UGT1A9 as dapagliflozin 3-O-glucuronide. Empagliflozin, which is 86% plasma protein bound, has a bioavailability of 90%. This molecule has T½ of about 14–18 h and gets eliminated by glucuronidation. Ertugliflozin is 93% plasma protein bound, and it has 100% bioavailability. Its t½ is 16 h, and it is metabolized by UGT1A9- and UGT2B7- mediated O-glucuronidation to inactive metabolites. In terms of affinity, all these agents show greater preference for SGLT2 over SGLT1, and tofogliflozin is the most selective for SGLT2. This agent binds SGLT2:SGLT1 with an 18-fold increase in selectivity compared to canagliflozin (2912:1 & 155:1, respectively). Canagliflozin shows the lowest oral bioavailability (F = 68%), while ertugliflozin possesses the highest F = 1 at doses of 15 mg. In general, this drug class is (generally) highly protein-bound to albumin, mainly excreted as O-glucuronide metabolites in the feces and urine, and has similar oral side-effect profile.

Canagliflozin was the first approved agent in the United States, and bexagliflozin is the most recently approved drug under this class. They exert a hemodynamic effect through osmotic diuresis, reducing glucose reabsorption, lowering renal glucose thresholds, and increasing urinary glucose excretion. Urinary glucose excretion is increased by 60–80 g per day, representing a loss of 240–320 kcal, promoting weight loss. These agents decrease hemoglobin A1c by an average of 0.6–0.8% in patients with type 2 diabetes; the glucosuria and subsequent natriuresis confer cardioprotective benefits in patients with and without diabetes, such as lowering both pre-and afterload of the heart and suppressing sympathetic activity. A meta-analysis of 45 trials showed an average reduction in HbA1c of 0.79% (monotherapy), 1.7 kg loss in body weight (~2.4%), 4/2 mmHg drop in blood pressure, and an increase in serum HDL by 6–9% [27].

Adverse effects mostly involve genitourinary tract infections, hypovolemia, some gastrointestinal (GI) disturbances, constipation, and diuresis. The female patients showed more susceptibility to genital infection [27]. This was evident in the EMPA-REG trial, where women in the empagliflozin treatment group displayed genital infections more frequently compared to men. Two other frequently encountered adverse effects were Fournier′s gangrene and diabetic ketoacidosis [28,29]. Bersoff-Matcha et al. reported a link between the use of SGLT2 inhibitor and Fournier’s gangrene, where they detected 55 cases over a six-year period [28]. Although a decreased blood pressure may be advantageous in many type 2 diabetic patients, hypovolemia could be challenging for them. That is why the prescribing information of these agents carries a warning asking for the evaluation of volume status before starting and during therapy. In terms of the development of diabetic ketoacidosis, the suggested mechanism includes a diminished insulin secretion with a concomitant increase in the synthesis of fatty acids that are converted to ketone bodies. Although canagliflozin had a black box warning for amputation risk, US FDA removed this after a thorough review of fresh clinical trials.

Initially, there was a concern regarding an increased risk of bladder cancer with the use of SGLT2 inhibitors due to constant exposure of the urinary tract to glycosuria, deterring their potential novel applications [30]. However, recent meta-analyses on randomized control trials have negated any significant difference with regard to malignancies (all types) or bladder cancer (specifically) in patients treated with SGLT2 inhibitors compared to other hypoglycemic agents or placebos. Despite studies suggesting SGLT2 inhibitors have no significant effect on new tumor incidences, longer-term studies should be conducted since most research was limited to a few years, retrospectively.

**Table 1:** Pharmacokinetic and pharmacodynamic properties of SGLT2 inhibitors. Compiled from [26,27,31,32,33,34,35,36,37].

Drug Name	SGLT2/1 Affinity	Absorption	Distribution	Metabolism	Elimination	Adverse Effects
Bexagliflozin	TBD	F: 78%, PPT: 2–4 h	93% bound, Vd: 262 L.	Glucuronidation (minor CYP role)	T1/2: 12 h Clearance: 19.1 L/h Feces: 51.1% Urine: 40.5%	Female genital mycotic infections, UTI, and increased urination
Canagliflozin	155:1	F: 65%, PPT: 1–2 h	99% bound, Vd: 119 L	Glucuronidation, (minor CYP role)	T1/2: 12 h Clearance: 192 mL/min Feces: 51.7% Urine: 33%	Genital mycotic infections (>10% [women]) UTI, thirst, constipation, volume depletion
Dapagliflozin	1242:1	F: 78%, PPT: 2 h	91% bound	Glucuronidation, (minor CYP role)	T1/2: 8–12 h Feces: 21% Urine: 75%	Renal adjustments for >65YO & eGFR 30–60 mL/min, nasopharyngitis, UTI, back pain
Empagliflozin	2680:1	F: 90% PPT: 1.5 h AUC_SS_: 1870 nmol*h/L	86.2% bound, Vd: 73.8 L	Glucuronidation	T1/2: 14–18 h Clearance: 10.6 L/h Feces: 41.2% Urine: 54.4%	UTI, URTI, diuresis, arthralgia, (postmarket) angioedema & AKI
Ertugliflozin	2235:1	F: 100% PPT: 1 h AUC_SS_: 398 ng*h/mL	93.6% bound, Vd: 85.5 L	Glucuronidation,	T1/2: 16.6 h Clearance: 11.2 L/h Feces: 40.9% Urine: 50.2%	Female: Genital mycotic infections (>10%), vaginal pruritus, Volume depletion, UTI, HA (postmarket) Fournier’s Gangrene
Ipragliflozin *	254:1	PPT: 2.6 ± 1.3 h AUC 27,299 ± 4622	TBD	Glucu- ronidation	T1/2: 10.3 ± 1.6 Low urinary excretion ~1%	GI disorders
Luseogliflozin *	1770:1	Tmax 0.5 (0.5–1.0) h AUCinf(ng·h/mL) 2010 ± 508	TBD	CYP-mediated metabolism	T1/2: 10.4 ± 0.832 h	Minimal AE, No UTI (mostly male patients)
Tofogliflozin *	2912:1	AUC0–24 h (h × ng/mL): 6 740 ± 1 680 Tmax (h) 0.750 (0.50–4.00)	TBD	CYP-mediated metabolism (CYP2C18, 4A11, and 4F3B)	T1/2: 3.98 ± 0.520 h	Increase in blood ketone body

* Not currently approved by US FDA; Vd: Volume of distribution; UTI: Urinary tract infection; URTI: Upper respiratory tract infection; eGFR: Estimated glomerular filtration rate; AKI: Acute kidney injury; AUC: Area under curve; TBD: To be determined; PPT: Peak plasma time.

## 5. Current Status of SGLT2 Inhibitors in Cancers

### 5.1. Anticancer Potential of SGLT2 Inhibitors in Cancers

Accumulating evidence has reported that SGLT2 inhibitors may be effective in eliminating tumor cells. Their anti-proliferative activity has been broadly attributed to the attenuation of glucose uptake in different in vitro and in vivo models (Table 2). Additionally, the favorable outcome with SGLT2 inhibitors in malignancies includes metabolic reprogramming, lowering of inflammation, and decreasing oxidative stress [38]. Villani et al. reported that canagliflozin in concentrations of 5–30 µM inhibited the proliferation and clonogenic survival of breast cancer cells (MCF7) [39]. They revealed that canagliflozin inhibited the mitochondrial complex-I that ultimately rendered the anti-lipogenic and anti-proliferative effects. This resulted in a disruption in cellular respiration and a significant increase in 5′ adenosine monophosphate-activated protein kinase (AMPK) activity [39]. Zhou et al. have shown that SGLT2 inhibitors, namely, dapagliflozin and canagliflozin promoted AMPK-mediated cell cycle arrest and apoptosis in MCF-7 breast cancer cells [40]. These agents downregulated oxidative phosphorylation, lowered intracellular ATP concentration, and upregulated AMPK phosphorylation in a dose-dependent manner and elicited mammalian target of rapamycin (mTOR) blockade. The researchers are actively seeking a potential treatment for breast cancer through mTOR suppression. Based on this study, SGLT2 inhibitor-induced cell cycle arrest in G1/G0 phase and apoptosis [40]. In another study, Komatsu et al. showed the dose-dependent anti-proliferative effect of ipragliflozin in MCF-7 cells [41]. This anti-proliferative effect was due to the inhibition of both glucose and sodium influx. Moreover, ipragliflozin was able to induce hyperpolarization of the cell membrane and mitochondrial membrane instability by blocking sodium influx [41]. Papadopoli et al. demonstrated that canagliflozin and dapagliflozin were able to block the proliferation of SKBR3 and BT-474 breast cancer cells, and this action did not depend on the intracellular glucose level [42]. Canagliflozin also interfered with cellular respiration and ATP production by disturbing glutamine utilization which is now claimed to be an important mechanism for the anti-proliferative action of this drug in breast cancer cells [42]. 

Li et al. reported canagliflozin-mediated apoptosis in non-small cell lung cancer that was independent of SGLT-2 inhibition. Canagliflozin inhibited L858R/T790M EGFR kinase anticancer effects in lung cancer cells that were resistant to EGFR TKIs [43]. A study conducted by Yamamoto et al. also supported the efficacy of canagliflozin in lung cancer. Specifically, in this study, canagliflozin attenuated the growth of A549 cells in a dose-dependent manner and inhibited S phase entry of A549 cells by restraining cell cycle progression [44]. SGLT2 inhibitors are also useful against osteosarcoma, as evidenced by Wu et al. In their study, they found that canagliflozin significantly inhibited osteosarcoma tumor growth with a concomitant infiltration of immune cells in vivo [45]. This drug stimulated STING expression and activated the IRF3/IFN-β pathway, which eventually suppressed AKT phosphorylation. Canagliflozin also exerted synergistic antitumor effects in combination with the STING agonist 2′3′-cGAMP [45]. Xu et al. showed a favorable outcome with canagliflozin in pancreatic cancer where this drug dose dependently repressed the growth of Capan-1 and PANC-1 cells. This drug also resulted in a 45.2% reduction in PANC-1-derived tumors in nude mice. This agent furnished a greater efficacy when combined with gemcitabine in Capan-1 and PANC-1 cells compared to treatment with gemcitabine alone. The authors reported that the canagliflozin-induced antitumor effect was due to a decrease in glucose transporter-1 and lactate dehydrogenase A [46].

Wang et al. revealed attenuation of thyroid cancer cell growth in vitro and in vivo via blockade of SGLT2 [47]. In this study, canagliflozin blocked glucose uptake, suppressed glycolysis and AKT/mTOR signaling activation, and increased AMPK activation in thyroid cancer cells. The drug also inhibited the G1/S phase transition and increased apoptosis [47]. Nakano et al. analyzed the effect of canagliflozin on the growth and metabolic reprogramming of hepatocellular carcinoma (HCC) using multi-omics analysis of metabolomics and absolute quantification proteomics [48]. They found that canagliflozin significantly inhibited the proliferation of Hep3B and Huh7 cells in a mechanism independent of the inhibition of the SGLT2-mediated glucose influx [48]. Hung et al. showed that canagliflozin blocked the translocation of β-catenin from the cytoplasm to the cell nucleus, which was partially independent of SGLT2 [49]. In fact, the proteasomal degradation of β-catenin took place by direct inhibition of protein phosphatase 2A (PP2A) activity [49]. Meanwhile, Kaji et al. reported that canagliflozin inhibited the proliferation of Huh7 and HepG2 cells in a dose-dependent pattern and significantly reduced their intracellular ATP levels. Finally, canagliflozin also induced apoptosis in these cells via caspase3 activation [50]. Villani et al. also reported that canagliflozin suppressed cellular proliferation and clonogenic survival of PC-3 prostate cancer cells alone and in combination with ionizing radiation and docetaxel. Mechanistically, canagliflozin decreased mitochondrial complex-I supported respiration and increased the phosphorylation of AMPK [39].

Interestingly, SGLT2 inhibitors have certain non-canonical effects that could contribute to the anticancer effects in a glucose-independent manner. SGLT2 inhibitors reduce the expression of angiotensin and promote osmotic diuresis by upregulating plasma renin [51]. This results in a reduction in blood pressure and increased urinary sodium excretion [52]. These drugs also inhibit myocardial Na+/H+ exchange (NHE), which leads to an increase in sodium ions in mitochondria in patients with heart failure. Furthermore, SGLT2 inhibitors are associated with improved myocardial metabolism and can upregulate myocardial oxygen supply, increase oxygen uptake, enhance ketone bodies, and shift from glucose to ketone utilization during myocardial metabolism [53]. These properties of SGLT2 inhibitors may play important roles in their anticancer effects. Similarly, the sympatholytic properties of SGLT2 inhibitors can attenuate sympathetic hyperactivity in various cancers. Balcıoğlu et al. showed that dapagliflozin was associated with improved cardiac autonomic function in type 2 diabetic patients that had cardiac autonomic neuropathy [54]. Another study by Lymperopoulos et al. also revealed that dapagliflozin elicited sympatholysis, even in non-diabetic heart failure patients. This study lends support to the fact that the cardiovascular benefit of dapagliflozin was independent of its blood glucose-lowering effect [55]. Such type of sympatholytic effect could be instrumental in the SGLT2 inhibitor-mediated anticancer effects since a very recent report demonstrated that suppressing β2-adrenergic signaling in the tumor microenvironment potentiated the efficacy of doxorubicin in a triple-negative breast cancer model [56]. Anthracycline chemotherapy promoted sympathetic activity and increased the concentration of norepinephrine in mammary tumors. The obstruction of sympathetic signaling resulted in the suppression of metastasis in mouse xenografts [56]. Thus, it is conceivable that supplementing SGLT2 inhibitors with traditional chemotherapeutics might be beneficial in the treatment of cancer, where SGLT2 inhibitors can not only block the supply of glucose, the primary fuel for the tumor cells but also curtail sympathetic output.

**Table 2:** Anticancer activities of SGLT2 inhibitors. Compiled from [39,40,41,42,43,44,45,46,47,48,49,50,57].

Tumor Site	Cancer Model	SGLT2i for Intervention	Study Type	Results
Breast	MCF-7 (human)	Canagliflozin	in vitro	Inhibition of cell proliferation, clonogenic survival, and colony formation
	MCF-7 (human)	Canagliflozin and Dapagliflozin	in vitro/in vivo	Significant reduction in tumor volume; Potently inhibited cell proliferation; Induced cell cycle arrest and apoptosis
	MCF-7 (human)	Ipragliflozin	in vitro	Inhibition of glucose and sodium influx; Alteration in mitochondrial membrane potential
	SKBR3, BT-474, MCF-7 (human)	Canagliflozin	in vitro	Inhibition of oxygen consumption and glutamine metabolism
Liver	Huh7, HepG2 (human)	Canagliflozin	in vitro	Inhibition of glucose uptake
	Huh7, Hep3B	Canagliflozin	in vitro/in vivo	Inhibition of β-catenin pathway
	Humans	Canagliflozin	*Patient sample*	suppression of angiogenesis
	Huh7, Hep3B	Canagliflozin	in vitro	Downregulation of ATP synthase F1 subunit alpha (mitochondrial electron transport system protein)
Pancreas	Capan-1, PANC-1	Canagliflozin	in vitro/in vivo	Suppression of glucose transporter-1 and lactate dehydrogenase A
Thyroid	TPC-1, BCPAP, Nthy-ori-3–1	Canagliflozin	in vitro/in vivo	Inhibition of glucose uptake, glycolysis, and AKT/mTOR signaling activation; increased AMPK activation
Bone	MNNG/HOS, MG-63, 143B, U2OS, K7M2	Canagliflozin	in vitro/in vivo	Activation of STING/IRF3/IFN-β pathway and suppression of AKT phosphorylation
Lung	A549 (human)	Canagliflozin	in vitro	Inhibition of cell cycle progression
	HCC827, H1975	Canagliflozin	in vitro	Inhibition of EGFR kinase
Prostate	PC3	Canagliflozin	in vitro	Inhibition of complex I supported mitochondrial respiration

### 5.2. Molecular Mechanisms of Anticancer Activities

**Figure 2:**
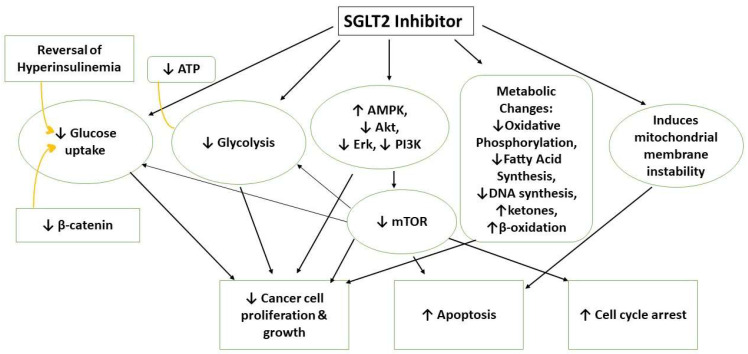
Mechanisms of anticancer effects of SGLT2 inhibitors.

Creating more effective and targeted therapeutics to palliate cancer symptoms, reduce tumor size, and deprive transformed cells of their energy supply requires an understanding of rewired metabolism of cancer (Figure 2). Taking advantage of mutated, altered, or deleted metabolic enzymes in cancer cells presents an opportunity for inhibition of its bioenergetics and growth. Although currently, the reports on the mechanistic aspect of SGLT2 inhibitors in suppressing tumor growth are limited, several studies revealed that suppression of glutamine metabolism and interference with several signaling cascades (such as PI3K/AMPK) might be crucial behind their anticancer effects [42].

Papadopoli et al. showed that a combination of canagliflozin and metformin had synergistically enhanced anti-proliferative effects on cancer cells by blocking mitochondrial complex I (MCI) [42]. However, recent studies revealed that canagliflozin (independent of SGLT expression levels and glucose availability) reduces cellular respiration activity in breast cell cancer by inhibiting glutamine metabolism via glutamine dehydrogenase (GLDH). Increased GLDH activity levels aid neoplastic proliferation by conferring adaptations to metabolic stress through favorable regulation of redox homeostasis, effects linked to poor cancer prognosis [42].

The metabolic rewiring undergone by cancer cells is also linked to an overexpression of several oncogenic signaling pathways. Researchers used canagliflozin and gamma-irradiation (γ-IR) to treat hepatocellular (HEP2) cancer cells. They measured the expression levels of various signaling pathways (PI3K/AKT/GSK3-β/mTOR and Wnt/β-Catenin) and apoptotic markers. Post-treatment, low expression levels revealed inhibitory activity throughout these cascading pathways essential for metabolic rewiring during tumor progression [58]. Canagliflozin and γ-IR inhibited glucose uptake and anaerobic glycolysis and induced endoplasmic reticulum autophagy. Another study on breast cancer found that the antineoplastic and anti-proliferative potential of canagliflozin was due to decreased levels of cellular ATP and AMPK activation [11]. Increased activated AMPK inhibits cellular protein synthesis and proliferation, causing cell cycle arrest and apoptosis in the G1 phase. Increased AMPK activation, inhibition of AKT/mTOR, and suppressed cyclin levels were also discovered in thyroid cancer cells treated with canagliflozin [47].

### 5.3. Clinical Evidence of SGLT2 Inhibitors in Cancer

The potential clinical success of SGLT2 inhibitors as anticancer agents is currently being evaluated in different clinical trials. A search on ClinicalTrials.gov with the keywords “cancer, sodium-glucose cotransporter 2 inhibitors” or “cancer, SGLT2 inhibitors” yielded ten studies with humans. Among those, three were observational in nature, and seven were interventional studies.

Other than the two completed studies, seven studies are currently recruiting participants, and one study is scheduled to be completed by June 2023. The first human pilot trial with 15 participants assessed the tolerability and efficacy of dapagliflozin (in addition to standard care) in patients with metastatic pancreatic ductal adenocarcinomas. Research outcomes involved tolerability of treatment, effects on plasma glucose, changes to computed tomography quantified tumor size, and tumor necrosis. Researchers hypothesized that treatment with dapagliflozin would be efficacious as an adjunct to frontline chemotherapy due to its metabolic pleiotropic effects [59]. The second trial, which had an estimated completion time in December 2021, is a phase 1b/2 interventional study involving 60 participants with advanced solid tumors (breast, endometrial, lung, colorectal, and head and neck cancers) treated in combination with serabelisib (PI3K inhibitor) and canagliflozin. The study aimed to see if the glucose/insulin feedback pathway will enhance the efficacy of PI3K inhibition. Primary outcomes include rates of adverse events, lab abnormalities, and dose confirmations, while secondary outcomes are tailored to the pharmacokinetic (PK) profile of serabelsib (Cmax, Tmax, AUC) [60].

Three other trials are currently in the recruitment stage or final stages of completion and merit acknowledgment. These trials share some overlap in drug selection and tumor selection with the plan to assess the efficacy of dapagliflozin (with or without metformin) in combination with alpelisib and fulvestrant in the treatment of advanced breast cancer [61,62,63]. The primary outcome will assess the reduction in all-grade hyperglycemia, while secondary outcomes will measure progression-free survival, overall response rate, and clinical benefit rate. Another phase I interventional study is currently recruiting participants with pediatric brain tumors for treatment with dapagliflozin and carmustine [64]. A phase I interventional trial involving 24 participants is planning to assess the tolerability/efficacy of neoadjuvant SGLT2 inhibition before radical prostatectomy in high-risk localized prostate cancer. The researchers hypothesize that four weeks of treatment with dapagliflozin will be safe and efficacious in these patients [65].

The results from limited preclinical studies indicate the effectiveness of SGLT2 inhibitors in decreasing the risk of certain cancers in humans. The completed phase 1b study by Park et al. (2023) suggests that dapagliflozin has tumor-suppressive effects in pancreatic ductal adenocarcinoma, with no new or severe adverse effects observed [66]. Another case report explored the role of canagliflozin in a patient with type 2 diabetes and hepatocellular carcinoma. The treatment plan included radiofrequency ablation, transcatheter arterial chemoembolization, and the SGLT2 inhibitor for glycemic control following metformin and glimepiride. After ten weeks of canagliflozin (100 mg/day) use, elevated serum alpha-fetoprotein levels normalized in addition to regression of hepatocellular carcinoma and negative tumor staining [57]. These results are possibly due to improvement in insulin resistance in the patient and downregulation of angiogenesis in hepatocellular carcinoma [11].

## 6. Conclusions

Despite substantial advancement in cancer treatment, it is still the second leading reason of death worldwide. Since SGLT2 is known to be expressed in different cancers, their inhibitors hold an immense potential to be used in this lethal ailment. Furthermore, since their glucose-lowering effect is independent of insulin action, this might be advantageous. The exact anticancer mechanisms of SGLT2 inhibitors are yet to be delineated. However, the inhibition of glucose transport along with a disruption in mitochondrial membrane potential, downregulating β-catenin and PI3K/Akt/mTOR pathways, priming of apoptosis etc., are a few reported mechanisms of these agents.

Targeting glucose transporters creates a potential avenue to starve cancer cells and reduce tumor size/progression while avoiding the undesirable side effects of current chemotherapy treatments. Over time, SGLT2 inhibitors have evolved as anticancer agents that displayed favorable results in different cancer models. By preventing glucose reabsorption and promoting urinary glucose excretion, SGLT2 inhibitors deplete energy stores for cancer cells, causing a rapid reduction in angiogenesis and environmental viability. Nevertheless, studies also showed that the anticancer effects of these agents are independent of their glucose-lowering effects as well. Pretreatment with SGLT2 inhibitors or even combining these agents with already established chemotherapeutics could be a viable option to investigate their additive or synergistic potential in oncology. Additionally, diabetic patients who concomitantly have cancer might also benefit by having these agents in their therapy regimen, although clinical trials are necessary to confirm this. Though clinical trials are mostly novel and ongoing, harnessing the pleiotropic effects of SGLT2 inhibitors creates new avenues for research and insight into evolving cancer therapies that may reduce tumor growth and impart diabetic and cardiac profiles while improving patient outcomes. However, the most crucial limitations of these agents as of now are their safety and clinical feasibility in cancer treatment. Therefore, more comprehensive research or clinical trial is indispensable to explore these exciting novel antidiabetic agents in cancer treatment.

## Data Availability

Not applicable.
